# Effects of Curcumin and Estrogen Receptor Alpha in Luminal Breast Cancer Cells

**DOI:** 10.3390/diagnostics14161785

**Published:** 2024-08-16

**Authors:** Lorena Palacios-Navarro, Leodan A. Crispin, Juan P. Muñoz, Gloria M. Calaf

**Affiliations:** Instituto de Alta Investigación, Universidad de Tarapacá, Arica 1000000, Chile; lorena.palacios@ug.uchile.cl (L.P.-N.); kcrispi@gestion.uta.cl (L.A.C.); jpmunozb@academicos.uta.cl (J.P.M.)

**Keywords:** curcumin, breast cancer, estrogens, estrogen receptor alpha, 17ß-estradiol, antiestrogens, cancer therapy

## Abstract

This work examined the potential benefit of curcumin in breast cancer patients as a supplementary drug in ER-positive cancers. The results indicated that in the MCF-7 human breast cancer cell line, E2 and curcumin decreased cell proliferation and the colony-forming capacity and down-regulated protein expression as well as important molecules associated with cell proliferation, such as PCNA and estrogen receptor alpha; genes associated with the epithelial-mesenchymal transition, such as β-catenin, Vimentin, and E-cadherin; and molecules associated with apoptosis. Clinical studies in bioinformatics have indicated a positive correlation between *ESR1* and either *CCND1* or *BCL2* gene expression in all breast cancer patients. Thus, curcumin could become a potential natural adjuvant treatment for patients with estrogen receptor alpha-positive breast cancer and those with resistance or a poor response to endocrine therapy since the reactivation of estrogen receptor alpha is inevitable.

## 1. Introduction

Breast cancer is the most common malignancy in women around the world and the leading cause of cancer-related death [1]. According to the WHO, this disease reached about 2.3 million cases in 2020, and over 500,000 death cases are reported each year [2]. About 80% of breast cancer cases diagnosed are ER-positive [3], i.e., they overexpress estrogen receptors (ERs) in the malignant tissue. Therefore, ERs have become a key target for developing therapies [4].

Several types of therapies have been developed to treat estrogen receptor alpha (ERα)-positive breast cancer. These therapies consist of administering drugs to prevent hormones such as 17β-estradiol (E2) from binding to their receptors (i.e., ERα) and enhancing tumor and/or cancer growth. These treatments specifically directed against ERα activity are called endocrine therapy [5]. Endocrine therapy is usually administered for 5 to 10 years and has become the main adjuvant treatment for ERα-positive breast cancer [6].

Medicinal plants are natural alternatives that have shown great potential in the treatment of diseases such as cancer [7]. Among them is curcumin (CUR) (1,7-bis(4-hydroxy-3-methoxyphenyl)-1,6-heptadiene-3,5-dione), which is extracted from the Curcuma longa plant and is commonly used in the preparation of curries in Asian countries for its color and flavor. CUR exerts anti-proliferative and apoptotic effects and has antioxidant properties. Due to these characteristics, it has been proposed as a chemo-preventive agent in cancer for clinical oncological use [8].

The polyphenolic bioactive compounds identified in turmeric are termed curcuminoids, the most abundant of which is CUR, which has been a target of study over the past few decades due to its therapeutic potential in addition to being a chemo-preventive, anti-inflammatory, anti-proliferative, and anti-carcinogenic agent [9]. Due to its great molecular complexity, CUR has been shown to modulate multiple signaling pathways, such as those involved in apoptosis, cell survival, tumor suppression, and caspases in death receptor pathways [10], such as the PI3K/Akt, MAPK, and NF-*k*B pathways in breast cancer [11,12]. Previous studies revealed the anti-proliferative actions of CUR, which rely on the presence of ERs in breast cancer cell lines [13,14]. According to reports, ERα expression is down-regulated in breast cancer cells treated with CUR compared with control cells [15]. For the latter reason, it is interesting to analyze the effect that CUR has on ERα activity. The reactivation of ERα independently of E2, the main mechanism of resistance to endocrine therapy, may occur through the interference of Erα with these oncogenic signaling pathways [16,17].

It is known that CUR modulates oncogenic signaling pathways and that it has multiple suppressive effects on ERα-positive malignant breast cell lines [14]. This study aimed to evaluate the effect of CUR and E2 in the MCF-7 human breast cancer cell line on cell proliferation, anchorage independence, and BCL2 and BAX protein expression; the expression of genes associated with the epithelial-mesenchymal transition, such as β-catenin and Vimentin; and other estrogen-responsive genes such as *cyclin D1*, *EGFR*, *cathepsin D*, and *BCL2* expression levels in comparison with the control, and is to be complemented by bioinformatics from clinical studies in breast cancer patients.

## 2. Materials and Methods

### 2.1. Chemical Reagents

Dimethyl sulfoxide (DMSO) was used to dissolve curcumin (CUR) (Sigma-Aldrich, St. Louis, MO, USA; CAS number 458-37-7, purity > 65%). A stock solution was created at a concentration of 100 M, filtered, aliquoted, and kept at 4 °C in the dark. 17α-estradiol (E2) was obtained from Sigma Aldrich (St. Louis, MO, USA), and analytical-grade ethanol was used to dissolve it at concentrations of 10 μM, 100 μM, and 10 mM. To achieve a concentration of 10 μM, MG132 (Cell Signaling Technology, Danvers, MA, USA) was reconstituted in DMSO.

### 2.2. Cell Line

The MCF-7 (ATCC) breast cancer cell line (#C0006008) (Addexbio, San Diego, CA, USA) was grown in DMEM (Gibco, Carlsbad, CA, USA), which contained 10% (*v*/*v*) fetal bovine serum (FBS) (Hyclone, Fremont, CA, USA) and 10 g/mL insulin (CAS 11061-68-0, Santa Cruz Biotechnology, Dallas, TX, USA). For the experiments, this cell line was grown in a hormone-reduced medium to eliminate steroid stimulation, DMEM devoid of phenol red, and supplemented with 5% charcoal-treated FBS, 1% sodium pyruvate, and 1% L-glutamine. A mixture of antibiotics, including 100 units/mL of penicillin and 100 g/mL of streptomycin, was added to all the cell media (Gibco, Carlsbad, CA, USA). All the cell cultures were kept in 75 cm^2^ Corning flasks (Tewksbury, MA, USA) at 37 °C in a humid environment with 5% CO_2_ saturation. After the cell adherence area filled 80% of the culture dish, the medium was changed after two to three days and passaged enzymatically by using trypsin and EDTA (0.05%).

### 2.3. Cell Cytotoxicity Assay

The cell cytotoxicity assay with MTT (3-(4,5-dimethylthiazol-2-yl)-2, 5-diphenyl tetrazolium bromide) was prepared as previously described [18]. The MCF-7 breast cell line was cultured in 96-well plates at a density of 5 × 10^3^ cells per well and incubated after 24 h with different concentrations of CUR and E2 for 48 h. Then, the cells were incubated with the MTT reagent (1:10 dilution in DMEM medium) at 37 °C for 4 h and treated with MTT in a DMSO solvent at 37 °C for 30 min. The formation of the formazan product was measured spectrophotometrically at 590 nm using a Multi-Modal Synergy HTX reader (Biotek, Winooski, VT, USA). The absorbance given is proportional to the number of live cells in each well.

### 2.4. Cell Viability with Crystal Violet Staining

After 48 h of incubation, the cells were shaken for 20 min at room temperature with a 0.5% crystal violet staining solution and each well of the plate was washed 4 times with distilled water and allowed to air dry without its lid for 2 h at room temperature. After drying, the cells were treated with methanol for 20 min at room temperature in an orbital shaker. The absorbance in each well was measured with a Multi-Modal Synergy HTX reader (Biotek, Winooski, VT, USA).

### 2.5. Growth in Soft Agar Assay Independently of Anchorage

The formation of the agar base was conducted as described in previous reports [18]. The cells were trypsinized, harvested, and re-suspended when the cultures had achieved 50% confluence, at a concentration of 10,000 cells/mL in a medium that included 10% FBS in DMEM supplemented with antibiotics. Subsequently, 2500 cells (0.25 mL) were amalgamated with 0.5 mL of the agar base, which was maintained at a temperature of 42 °C. This blend was then transferred to 35 mm dishes, which were pre-prepared with a solid layer of agar base. The cells were nourished bi-weekly with 0.5 mL of either full RPMI-1640 or DMEM medium, which contained CUR, E2, or an equivalent concentration of PBS as a control. This process was sustained for a month at a temperature of 37 °C in a CO_2_ incubator with a 5% concentration. After 35 days, the colonies were photographed.

### 2.6. Western Blot

The MCF-7 cell line was cultured in 6 cm dishes and grown for 2–3 days until 80% confluence. Following this confluence, the cells were treated with DMSO (control), E2 (1 × 10^−7^ M), CUR (25 μM), or CUR+E2 and left to incubate for 48 h. Then, the cells were lysed with 1X RIPA buffer supplemented with a 1X protease and phosphatase inhibitor (Roche Diagnostics, Basel, Switzerland) to determine the expression of PCNA, ERα, Bax, Bcl-2, β-catenin, and Vimentin. The Pierce BCA reagent (Thermo Fisher Scientific, Waltham, MA, USA) was used to determine the protein concentration according to the manufacturer’s instructions. Next, 20 μg of protein was denatured with a 1X loading buffer (National Diagnostics, Atlanta, GA, USA) at 95 °C for 5 min and then loaded onto a 1% sodium dodecyl sulfate-supplemented polyacrylamide gel (SDS-PAGE). The electrophoresis process was done at 100 Volts for two hours with a PowerPac power supply (Bio-Rad, Hercules, CA, USA). The gel proteins were then transferred onto a nitrocellulose membrane by employing the Trans-Blot^®^ SD semi-dry transfer apparatus at 20 Volts for 50 min, following the guidelines of the manufacturer (Bio-Rad, Hercules, CA, USA). Following this, the membrane underwent a blocking process involving a 5% solution of bovine serum albumin (BSA) and 0.1% of Tween 20 mixed in a tris saline buffer (TBS-T20) for 2 h at ambient temperature, followed by washing it thrice with TBS-T20, with each wash lasting for 10 min. The membrane then went through an incubation process with the primary antibodies for PCNA (sc-56), Bax (sc-526), Bcl-2 (sc-492), β-catenin (sc-1496), and Vimentin (sc-7557), all sourced from Santa Cruz Biotechnology, Inc., Santa Cruz, CA, USA, as well as ERα (D6R2W, Cell Signaling, Danvers, MA, USA), at a 1:1000 dilution in TBS-T20 overnight. Following the incubation period, the membrane underwent four wash cycles with TBS-T20, with each lasting 10 min. After this, it was exposed to a secondary antibody (BD Pharmingen, San Diego, CA, USA) diluted at 1:2000 in TBS-T20 for 2 h. The membranes were subsequently rinsed thrice in TBS-T20, with each rinse lasting for 10 min; then, following the manufacturer’s instructions, the peroxidase reaction signals were identified using the ECL system (Amersham Pharmacia Biotech, Little Chalfont, UK). An internal loading control, β-actin, was used to standardize the concentrations of all the proteins.

### 2.7. Real-Time PCR (RT-qPCR)

The MCF-7 cells were grown in 10 cm plates for 2–3 days until 80% confluence; treated with DMSO (control), E2 (1 × 10^−7^ M), CUR (25 μM), or CUR+E2 combined; and then incubated for 48 h. This technique was done according to previous studies [18]. Subsequently, the cells were broken down using 1 mL of TRIzol reagent, a product from Invitrogen (Thermo Fisher Scientific, Inc.). The RNA content was quantified using a Qubit^TM^ RNA Broad Range kit in conjunction with a Qubit 4.0 fluorometer, both of which were supplied by Thermo Fisher Scientific based in Waltham, MA, USA, as per the manufacturer’s guidelines. cDNA was generated using the AffinityScript kit from Agilent Technologies, Lexington, MA, USA, by following the instructions provided by the manufacturer. This cDNA then underwent quantification via real-time PCR (qPCR) on the CFX 96 Touch Detection System, a product of Bio-Rad Laboratories, Hercules, CA, USA, using specific primers. Each amplification blend was formulated with 12.5 μL of SYBR Green Mastermix 2X supplied by Promega (Madison, WI, USA), 0.4 μM of specific primers, and 1 μL of cDNA, all of which resulted in a total volume of 25 μL. qPCR was done for the genes *ESR1*, *CTSD*, *EGFR*, *CCND1*, *BCL2*, and *ACTB* (ß-actin) under the following thermocycling conditions: 94 °C for 30 s, 55 °C for 20 s, and 72 °C for 20 s, spanning 40 cycles. The primers for each pre-selected gene are outlined in Table 1. The experiments were conducted twice, and the cycle threshold was established using the BIO-RAD CFX Manager 2.1 program. The comparative gene expression was calculated using the 2^−ΔΔΔCt^ method, with the ß-actin levels acting as the base control.

### 2.8. Immunocytochemistry

To visualize the protein levels and the localization of ERα, PCNA, Bax, Bcl-2, β-catenin, and Vimentin, immunocytochemistry was conducted according to a previously described protocol [19]. When the cells reached 80% confluence, a total of 1 × 10^4^ cells were seeded onto a glass slide (courtesy of Nunc Inc., Naperville, IL, USA) in the company of DMSO (which served as the control), E2 (at a concentration of 1 × 10^−7^ M), CUR (at 25 μM), and a combination of CUR and E2. The cells were then cultivated for 48 h. Subsequently, the cells were stabilized at room temperature with buffered paraformaldehyde. This process was followed by inhibiting the natural peroxidase with a 1% solution of H_2_O_2_ in methanol. Afterward, the stabilized cells were rinsed twice again with PBS. Moving forward, the cell cultures were layered with regular horse serum for half an hour at room temperature. This was then followed by a procedure of incubation with primary antibodies, namely PCNA (sc-56), Bcl-2 (sc-492), Bax (sc-526), β-catenin (sc-1496), Vimentin (sc-7557), E-cadherin (sc-8426)—all of which were provided by Santa Cruz Biotechnology, Inc., Santa Cruz, CA, USA—and ERα (D6R2W, which was sourced from Cell Signaling, Danvers, MA, USA). The cells were then diluted to a ratio of 1:500 and incubated at 4 °C overnight. Following two PBS washes, the cells underwent a 45-min incubation period with a diluted solution of biotinylated secondary antibody and Vectastin Elite ABC reagent procured from Vector Laboratories Inc., located in Burlingame, CA, USA.

### 2.9. Gene Expression Analysis Using Bioinformatics

TIMER2.0, the Tumor Immune Estimation Resource v2.0 (http://timer.cistrome.org/), accessed on 6 August 2021, is an informational online repository that evaluates the clinical significance of various immune cells in different types of breast cancer through three main components—immune association, cancer exploration, and immune estimation. Each of these components contains unique modules that investigate the immunological, clinical, and genomic traits of tumors. As part of the research component, the Gene_Corr module showcases the relationships between the target gene and an array of genes in multiple breast cancer subtypes. The Gene_DE module within each breast cancer subtype highlights the genes that are either up- or down-regulated in tumors in comparison to normal tissues. The Gene_Outcome module reviews patients’ survival rates, adjusted by the clinical stage factor, across a range of breast cancer subtypes [20]. Given the inputs (for each module in the exploration component), TIMER2.0 generates a functional heatmap table that presents the association between each input feature, and detailed information about a relationship can be found intuitively by clicking on the corresponding entry. When the “purity adjusted” option is selected, clicking on any of the numbers in the table will return two scatter plots showing (i) the correlation of the given gene expression with the tumor purity (the proportion of cancer cells in a sample) and (ii) the association of the gene expression with the other input genes.

Through the UCSC Xena Functional Genomics Explorer (http://xena.ucsc.edu/) accessed on 20 August 2021, the University of California, Santa Cruz, provided the statistical significance of the correlation between genomic and/or phenotypic variables such as ER status [21].

### 2.10. Statistical Analysis

Gene correlations were assessed using the purity-adjusted Spearman’s rho test, the Wilcoxon rank-sum test was employed to examine the differential gene expression between tumors and the surrounding normal tissue, and the survival was assessed by utilizing the Cox proportional hazard model; these tests were estimated using TIMER2.0, reference number [20]. Via UCSC Xena, a One-way ANOVA was used to perform an estrogen receptor status analysis, reference number [21]. The data were analyzed with the GraphPad Prism v8 software (La Jolla, CA, USA) and are expressed as the average ± standard error of the mean. Comparisons between all the treated groups were made using an ANOVA and then Dunnett’s test to indicate statistical differences among the groups and the controls. A *p*-value of less than 0.05 was considered a difference of statistical significance.

## 3. Results

### 3.1. Cell Viability

**Figure 1:**
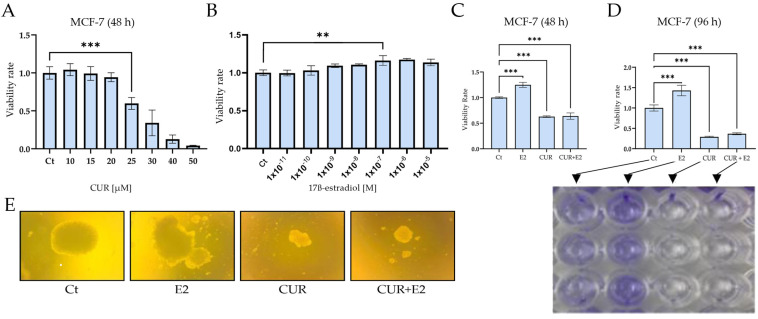
(**A**) The effect of 25 μM curcumin (CUR) for 48 h on cell viability in the MCF-7 human breast cancer cell line, an estrogen receptor-positive cell line. (**B**) 17ß-estradiol (E2) at 1 × 10^−7^ for 48 h. The viability was measured using (**C**) an MTT assay and (**D**) crystal violet staining, and DMSO was used as a control (Ct). (**E**) The effect of CUR, E2, and a combination of both on anchorage-independent growth in the MCF-7 cell line, measured using colony formation assays in semisolid agar for 31 days. Comparisons between all the treated groups were made using an ANOVA and then Dunnett’s test to indicate statistical differences among the groups and the controls (**A**–**D**). The data are expressed as the mean ± average with standard deviations (**: *p* < 0.01; ***: *p* < 0.001).

To analyze the viability rate of CUR and E2 in the MCF-7 cell line, the cells were exposed to different concentrations of CUR from 0 to 50 μM and E2 from 1 × 10^−11^ M to 1 × 10^−5^ M for 48 h (Figure 1A).

The MTT assay showed that the CUR viability increased dramatically (*p* < 0.001) between 10 and 25 μM compared to the control and that the IC50 was 25.7 μM after 48 h. Figure 1B shows that the E2 viability was significantly (*p* < 0.01) higher from 1 × 10^−11^ M to 1 × 10^−7^ M compared to the control after 48 h by using the same assay. Another viability assay was used to identify viable cells by staining with crystal violet to examine the effect of E2, CUR, and both substances combined. Both graphs showed that the cell viability significantly (*p* < 0.001) increased when the cells were exposed to E2 alone; however, CUR and the combination of CUR+E2 decreased the viability after 48 h (Figure 1C) and 96 h (Figure 1D) in comparison with the control. A colony formation assay is shown in Figure 1E and the results demonstrated that E2 increased, but CUR alone and the combination of CUR+E2 inhibited, the anchorage-independent growth or the loss of anoikis in MCF-7 cells.

**Figure 2:**
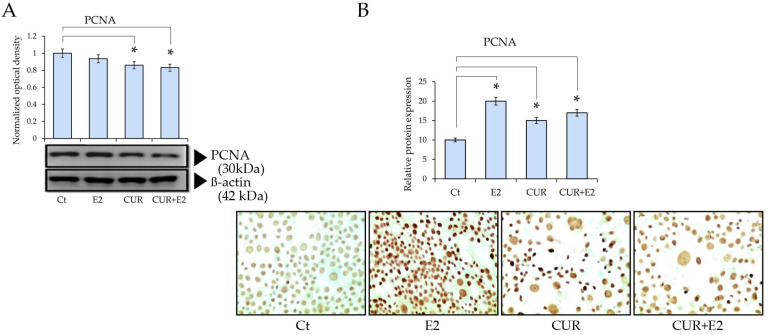
Effect of CUR, E2, and combination of both on proliferating nuclear antigen (PCNA) protein expression in MCF-7 cell line using (**A**) Western blot (β-actin served as control for loading) and (**B**) graph that represents PCNA protein expression according to relative peroxidase intensity in MCF-7 cell line, and its corresponding representative peroxidase images of immunocytochemistry analysis of PCNA (sc-56, Santa Cruz Biotechnology Inc., Santa Cruz, CA, USA) in MCF-7 cells treated with E2, CUR, or both combined for 48 h. Pictures were captured at 40× magnification using an Olympus CX31 optical microscope. Data are expressed as mean ± average with standard deviations (*: *p* < 0.05).

Cell proliferation protein levels were determined via Western blot, using the proliferating cell nuclear antigen (PCNA), as seen in Figure 2. There was a decrease caused by the CUR effect, both alone and combined with E2, in comparison to the control. This was confirmed using immunocytochemistry, which showed a decrease in the staining of cells under similar treatments.

### 3.2. Curcumin and Estrogen Receptor Alpha

**Figure 3:**
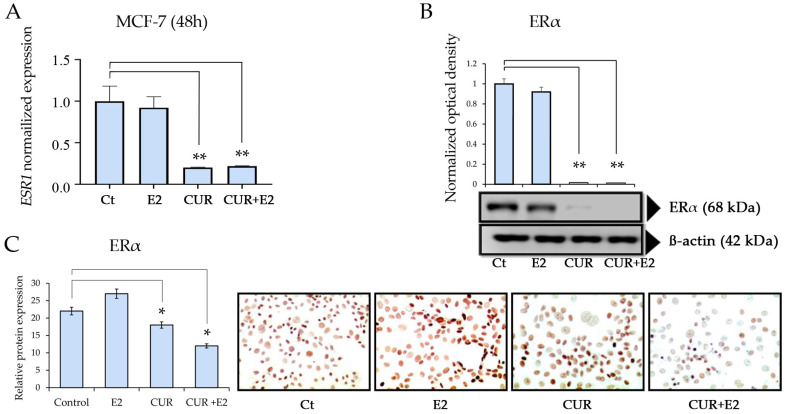
(**A**) The effect of curcumin (CUR) alone and in the presence of 17β-estradiol (E2) on ERα gene (*ESR1*) expression levels in the MCF-7 cell line. The cells were treated with DMSO as the control (Ct), E2, CUR, and CUR+E2 combined for 48 h. Comparisons between all the treated groups were made using an ANOVA and then Dunnett’s test to indicate statistical differences among the groups and the controls. (**B**) The effect of E2 and CUR, both alone and combined, on the ERα protein expression, with a Western blot and representative graphs, in MCF-7 cells treated for 96 h. β-actin was the loading control. (**C**) This graph represents the ERα protein expression according to the relative peroxidase intensity in the MCF-7 cell line and the corresponding representative peroxidase images for the immunocytochemistry of ERα (D6R2W, sourced from Cell Signaling, CA, USA) in MCF-7 cells. The images were taken with 40× magnification using an Olympus CX31 optical microscope. The data are expressed as the average with the standard deviation (*: *p* < 0.05; **: *p* < 0.01).

The effect of E2, CUR alone, and both combined were also analyzed on the *ESR1* gene expression levels after 48 h (Figure 3).

CUR and the combination of CUR+E2 significantly (*p* < 0.01) decreased gene expression, as seen in Figure 3A. Figure 3B shows the effects of E2, CUR alone, and both combined on the ERα protein expression levels. The results showed that CUR and the combination of CUR+E2 significantly (*p* < 0.01) decreased the protein expression in MCF-7 cells after 96 h.

### 3.3. Curcumin and Apoptotic Protein Levels

**Figure 4:**
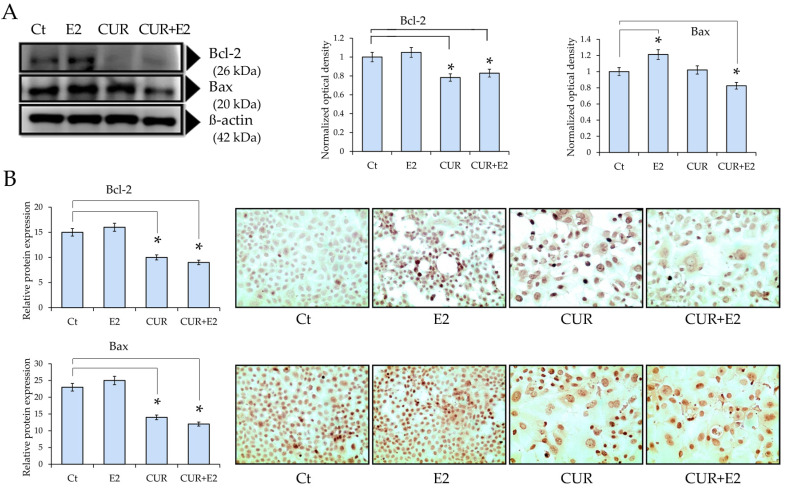
(**A**) The impact of curcumin (CUR) by itself and when combined with 17β-estradiol (E2) on the protein expression of Bcl-2 and Bax in the MCF-7 cell line, using a Western blot analysis and graphs for 48 h. DMSO was used as the control (Ct) and β-actin as the loading control. Comparisons between all the treated groups were made using an ANOVA and then Dunnett’s test to indicate statistical differences among the groups and the controls (*: *p* < 0.05). (**B**) The graphs represent Bcl-2 and Bax protein expressions according to the relative peroxidase intensity in the MCF-7 cell line and the corresponding representative peroxidase images of the immunocytochemistry of Bcl-2 and Bax (sc-492 and sc-526, respectively, both from Santa Cruz Biotechnology Inc., Santa Cruz, CA, USA) in the same cell line. The pictures were captured at 40× magnification using an Olympus CX31 light microscope.

The protein levels of apoptotic markers such as Bcl-2 and Bax in the MCF-7 cell line are shown in Figure 4.

The findings indicated that the Bcl-2 protein levels significantly (*p* < 0.05) decreased in cells exposed to CUR alone or CUR+E2 combined when compared to the control (Figure 4A). The Bax protein levels significantly (*p* < 0.05) increased in cells exposed to E2 in comparison with the control, whereas those levels decreased in response to the effect of CUR alone or CUR+E2 combined in comparison with the control, as shown via the Western blot and the corresponding graphs. Figure 4B shows representative images of the previous results.

### 3.4. Genes Affected by Curcumin and Estrogen

**Figure 5:**
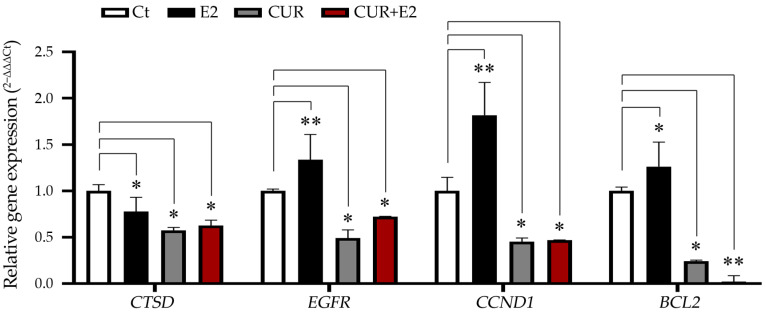
The effect of curcumin (CUR) alone and in the presence of 17β-estradiol (E2) for 48 h on the levels of the cathepsin D gene (*CTSD*), the epidermal growth factor receptor gene (*EGFR*), the cyclin D1 gene (*CCND1*), and the *BCL2* apoptosis regulator gene in MCF-7 cells, with DMSO as the control (Ct). The data are expressed as the average with the standard deviation. Comparisons between all the treated groups were made using an ANOVA and then Dunnett’s test to indicate statistical differences among the groups and the controls (*: *p* < 0.05; **: *p* < 0.01).

An investigation of gene expression in numerous processes within the MCF-7 cell line was conducted by examining the impact of E2, CUR, and the combined effect of E2+CUR (Figure 5).

The graph in Figure 5 shows that E2 significantly increased the expression of genes such as *EGFR* (*p* < 0.01), *CCND1* (*p* < 0.01), and *BCL2* (*p* < 0.05), while there was a decrease in the levels of *CTSD* (*p* < 0.05). CUR and the combination of CUR+E2 significantly (either *p* < 0.05 or *p* < 0.01) decreased the *CTSD*, *EGFR*, *CCND1*, and *BCL2* gene expressions.

### 3.5. Curcumin and Epithelial-Mesenchymal Transition (EMT)

**Figure 6:**
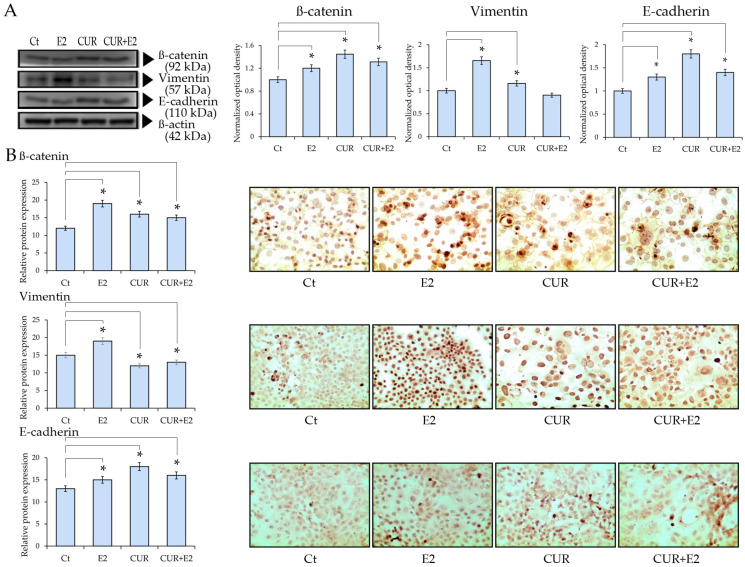
The effect of curcumin (CUR) alone and in the presence of 17β-estradiol (E2) on (**A**) the β-catenin, Vimentin, and E-cadherin protein expression in MCF-7 cells treated for 48 h. Western blot analysis: DMSO was used as a control (Ct) and β-actin was used as the loading control. Comparisons between all the treated groups were made using an ANOVA and then Dunnett’s test to indicate statistical differences among the groups and the controls (*: *p* < 0.05). (**B**) Graphs that represent ß-catenin, Vimentin, and E-cadherin protein expressions according to the relative peroxidase intensity in the MCF-7 cell line and the corresponding representative peroxidase images of the immunocytochemistry of β-catenin, Vimentin, and E-cadherin (sc-1496, sc-7557, and sc-8426, respectively; provided by Santa Cruz Biotechnology, Inc., Santa Cruz, CA, USA) in the same cell line. The images were taken with 40× magnification in an Olympus CX31 optical microscope.

The effect of E2, CUR, and their combination was evaluated to determine the EMT protein expression using a Western blot in the MCF-7 cell line, as seen in Figure 6.

The results demonstrated that E2, CUR, and the combination of CUR+E2 significantly (*p* < 0.05) increased β-catenin protein expression in comparison to the control. There was an increase in the Vimentin protein expression due to the effect of E2, whereas CUR, alone or combined with E2, decreased such an effect in comparison with the control. This is corroborated by the immunocytochemistry in Figure 6B, which shows representative images that indicate a pronounced staining for E2-treated cells, whereas CUR-treated cells had less intensity, with apoptotic cells among them.

### 3.6. Clinical Relevance in Breast Cancer Patients, Analyzed Using Bioinformatics

The Cancer Genome Atlas (TCGA), which was developed in partnership with the National Human Genome Research Institute (NHGRI) and the National Cancer Institute (NIH), has conducted the most exhaustive analysis across various cancers to date. They have created complex, multi-faceted maps detailing the significant genomic changes in 33 different cancer types [22].

#### 3.6.1. Gene Correlation in Breast Cancer Patients

**Figure 7:**
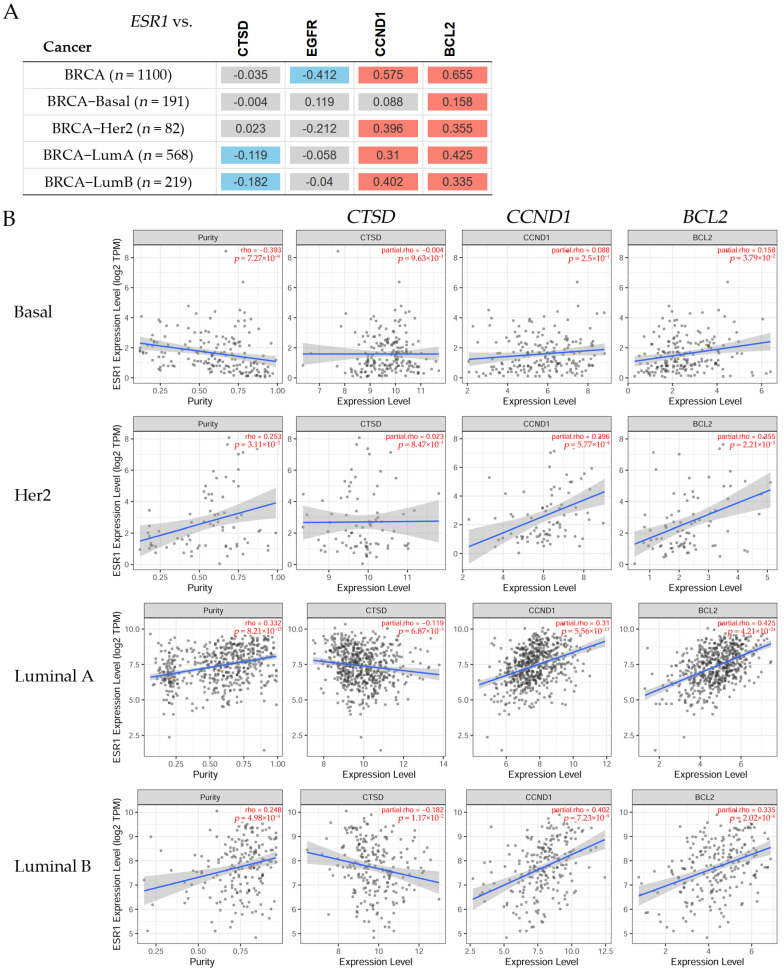
(**A**) The heatmap table shows the correlation between the estrogen receptor alpha gene (*ESR1*) and the cathepsin D (*CTSD*), epidermal growth factor receptor (*EGFR*), cyclin D1 (*CCND1*), and BCL2 apoptosis regulator (*BCL2*) gene expression levels in invasive breast carcinoma (BRCA) subtypes. The red color indicates a statistically significant positive correlation (Spearman’s, *p* < 0.05), the blue color indicates a statistically significant negative correlation (Spearman’s, *p* < 0.05), and gray denotes a non-significant result. (**B**) The scatter plots represent the significant (Spearman’s, *p* < 0.05) correlations between the expression of *ESR1* with a purity adjustment (on the left) and the expression levels of the *CTSD*, *EGFR*, *CCND1*, and *BCL2* genes (on the right) in patients with breast cancer. The correlation figures for each examination are indicated in red on the right side (adjusted partial Spearman’s rho value as the degree of their correlation). The expression level was estimated using TIMER2.0 (accessed on 17 June 2022) in breast cancer subtypes [20].

TIMER2.0 [20] was used to estimate the correlations between *ESR1,* with a purity adjustment, and the expression of *CTSD*, *EGFR*, *CCND1*, and *BCL2* in breast cancer subtypes (Figure 7).

The correlations between the *ESR1* and the *CTSD*, *CCND1*, and *BCL2* gene expression levels can be seen in Figure 7. There was no statistically significant difference between the *ESR1* and *CTSD* expression levels in patients with Basal or Her2 breast cancer (Figure 7A); however, there was a significant (*p* < 0.05) negative correlation in Luminal A and Luminal B patients. The correlation between the *ESR1* and *CCND1* expression was positive in Her2, Luminal A, and Luminal B patients, but not in Basal patients, where this correlation was non-significant. A significant (*p* < 0.05) positive correlation was found between the expression levels of *ESR1* and *BCL2* in Basal, Her2, Luminal A, and Luminal B patients. There was a non-significant difference between the *ESR1* and *EGFR* expression levels in any breast cancer subtype. Figure 7B shows scatter plots of *CTSD*, *CCND1*, and *BCL2* in Basal, Her2, Luminal A, and Luminal B patients. The results indicated that the correlation between *ESR1* and *CTSD*, *CCND1*, and *BCL2* had a partial rho that was very significantly low in Basal patients. Similarly, the partial rho was also very significantly low in Her2 patients. On the other hand, there was a negative correlation between *ESR1* and *CTSD* and its partial rho was significantly higher in Luminal A and Luminal B patients. The *CCND1* and *BCL2* expressions showed a positive correlation with *ESR1* and their partial rho values were significantly high in Luminal A and Luminal B patients.

#### 3.6.2. Variation in Gene Expression between Normal Tissues and Tumors across Different Breast Cancer Subtypes

**Figure 8:**
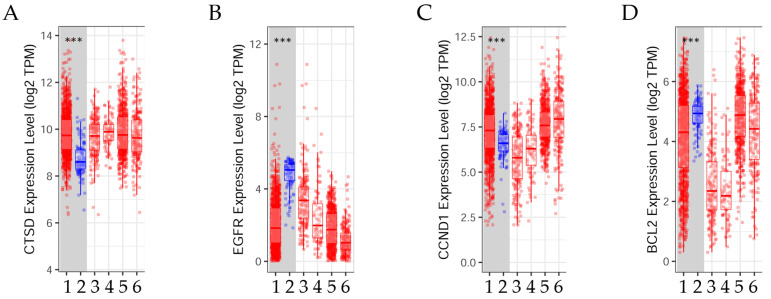
Variations in gene expression levels between tumor and normal tissues in invasive breast carcinoma across different subtypes. The box diagrams illustrate the (**A**) cathepsin D (*CTSD*), (**B**) epidermal growth factor receptor (*EGFR*), (**C**) cyclin D1 (*CCND1*), and (**D**) BCL2 apoptosis regulator (*BCL2*) gene expression levels in tumors compared to normal tissues (using the Wilcoxon rank-sum test, ***: *p* < 0.001). These levels were determined using TIMER2.0 (accessed on 17 June 2022) in patients with invasive breast carcinoma [20]. 1: Tumor (*n* = 1093), 2: Normal (*n* = 112), 3: Basal.Tumor (*n* = 190), 4: Her2.Tumor (*n* = 82), 5: Luminal A.Tumor (*n* = 564), 6: Luminal B.Tumor (*n* = 217).

The difference in gene expression between tumors and surrounding normal tissues in TCGA types of breast cancer can be seen in Figure 8.

The results indicated that the expression levels of the genes *BCL2* and *EGFR* were significantly (*p* < 0.001) higher in normal tissue than in tumor tissue. Conversely, the expression levels of the genes *CCND1* and *CTSD* were significantly (*p* < 0.001) elevated in tumors in contrast to normal tissues. These findings were determined through the use of TIMER2.0 in the analysis of invasive breast carcinoma [20].

#### 3.6.3. Gene Expression and Estrogen Receptor Status in Breast Cancer Patients

**Figure 9:**
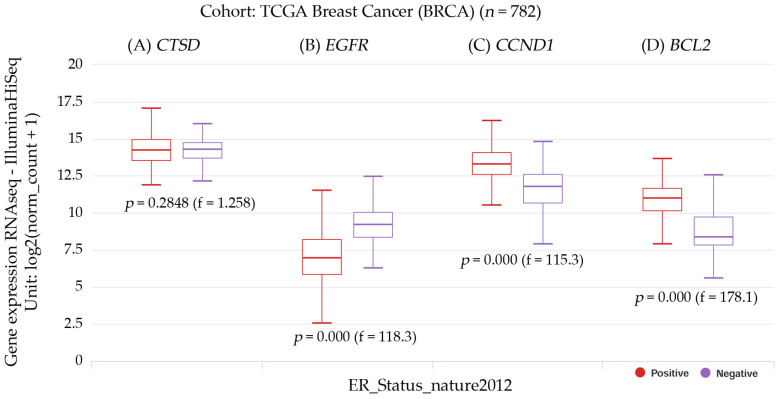
Box plot representations of the transcript expressions for the following genes: (**A**) *CTSD*, or the cathepsin D gene; (**B**) *EGFR*, or the epidermal growth factor receptor gene; (**C**) *CCND1*, or the cyclin D1 gene; and (**D**) *BCL2*, or the BCL2 apoptosis regulator gene, all within the context of invasive breast carcinoma. The studied cohort was TCGA breast cancer (BRCA), with 782 subjects, and it was stratified by nature2012 based on the estrogen receptor status. A one-way ANOVA test was used for the statistical analysis, with a *p*-value less than 0.05 considered significant. The information was sourced from the raw data obtained via the UCSC Xena functional genomics explorer from the University of California, Santa Cruz (https://xena.ucsc.edu/), accessed on 20 August 2022 [21].

Figure 9 illustrates the gene expression and estrogen receptor status of patients with breast cancer.

The transcript expressions of *CTSD*, *EGFR*, *CNND1*, and *BCL2* in breast cancer patients can be observed in the box plots. Patients with *CCND1* and *BCL2* gene expression had a significantly (*p* < 0.001) higher positive ER status. Patients with *EGFR* had a significant (*p* < 0.001) negative ER status, but those with high *CTSD* expression had a non-significant ER status. The results were estimated using UCSC Xena, reference number [21], accessed on 20 August 2022.

#### 3.6.4. Analysis of Gene Expression across Different Breast Cancer Subtypes Using Disease Stage Factor

**Figure 10:**
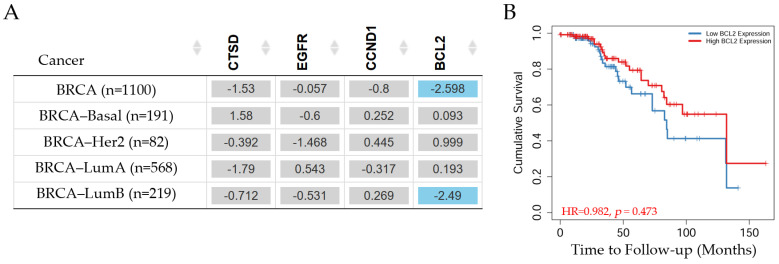
Survival in breast cancer patients. (**A**) The table depicts the normalized coefficient of the cathepsin D gene (*CTSD*), the epidermal growth factor receptor gene (*EGFR*), the cyclin D1 gene (*CCND1*), and the BCL2 apoptosis regulator gene (*BCL2*) in the Cox model, adjusted by clinical factors such as the stage in invasive breast carcinoma subtypes. The blue color indicates a statistically significant decreased risk (Z-score, *p* < 0.05) and gray denotes a non-significant result. (**B**) The Kaplan–Meier curve of the significant genes such as *BCL2* in Luminal B patients. These levels were determined using TIMER2.0 (accessed on 10 July 2024) in patients with invasive breast carcinoma, reference number [20].

Gene expression and patient survival data for different breast cancer subtypes are shown in Figure 10.

The survival of patients with invasive breast carcinoma can be seen in Figure 10. *CTSD*, *EGFR*, and *CCND1* gene expression did not show any significant differences in patients with breast cancer subtypes such as Basal, Her2, Luminal A, and Luminal B. However, *BCL2* showed a significant (*p* < 0.05) decreased risk in Luminal B patients according to the Cox proportional hazard model (survival analysis estimated using TIMER2.0). The Kaplan–Meier curve (Figure 10B) indicated that patients with high *BCL2* gene expression exhibited an approximate value of 0.3 on the cumulative survival axis and a decrease in the probability of survival by 70% around 130 months. Patients with low *BCL2* expression showed an approximate value of 0.18 in the cumulative survival axis at around 130 months.

The gene expression moderated by the disease stage factor analysis is illustrated in Table 2.

This research evaluated the clinical stages of patients suffering from invasive breast carcinoma, and the findings revealed that there was a significant (*p* < 0.001) increase in the expression levels of the *CTSD*, *EGFR*, *CCND1*, and *BCL2* genes, particularly in stage 4 in the case of the Her2, Luminal A, and Luminal B subtypes. However, in patients with the Basal subtype, there was no significant gene expression detected at any stage.

## 4. Discussion

It is estimated that approximately 80% of breast cancers present tumors that are ER-positive [3]; thus, these cancers are hormone-dependent. Such tumors require hormones, such as estrogens, to grow and are associated with cell proliferation and survival processes. Although E2 has beneficial properties for some organs of the human body, it is considered one of the major contributing risk factors for ER-positive breast cancer development and it was identified as a carcinogen by IARC in 2012 [23]. Based on the potential anticancer effect of CUR as previously demonstrated in malignant breast cell lines [24,25,26], this work considered the effect of E2 and CUR on the activity of ERα and other genes.

Radiation dermatitis is a frequent adverse effect experienced by numerous breast cancer patients undergoing radiotherapy; thus, the impact of curcumin on this was examined. As a result, various patient studies were conducted to assess the effect of curcumin on the severity of radiation dermatitis among breast cancer patients through randomized controlled trials. More specifically, the meta-analysis outcomes showed a significant decrease in the severity score of radiation dermatitis in the group that received curcumin supplements compared to the control group [27].

The present work demonstrated that E2 and CUR decreased the cell proliferation and colony-forming capacity, and down-regulated important molecules associated with ERs. The results also indicated that CUR alone and in the presence of E2 decreased the ERα levels after 48 h. Another study reported ERα degradation when cells were exposed to E2 for longer periods [28]. Both the ESR1 and ERα expression were higher in breast cancer tumors than in normal tissues, based on the differential gene expression in clinical patients. In breast cancer patients, the ESR1 gene expression was found to indicate a positive ER status. The results indicated that, in stages 3 and 4, the *ESR1* gene expression levels were highly expressed in all invasive breast carcinoma patients; however, in subtypes such as Her2, Luminal A, and Luminal B, they were only expressed in stage 4. In patients with the Basal subtype, there was no significant display of gene expression at any stage.

The results showed that the E2 levels increased the cathepsin gene, whereas CUR alone and in the presence of E2 decreased such levels after 48 h. Those genes and others had “estrogen response element sites” (ERE sites) in their regulatory sequences, such as the cathepsin D gene *CTSD* [29], which binds directly to ERα after activation. Contrary to normal cells, the majority of metastatic breast cancer cell lines express large amounts of pro-Cath-D. All tissues express the aspartyl lysosomal protease known as cathepsin D (Cath-D) and both the overexpression of the Cath-D gene and the altered precursor protein processing account for this abnormal secretion [30]. The transcription of the Cath-D gene in ER-positive breast cancer cells is stimulated by estrogen and growth factors, while an unknown process triggers the same activity in ER-negative cells.

An independent clinical study has linked the probability of future metastases to high Cath-D concentrations in the cytosol of primary breast tumors [30]. Some genes do not present these ERE sites (indirectly regulated genes), such as the gene coding for the *EGFR* gene. It is expected that the mRNA levels of ERα-regulated genes would increase in cells exposed to E2 and decrease in those exposed to CUR alone or in the presence of E2 [31]. In patients with Basal or Her2 breast cancer, no substantial connection was identified between the *ESR1* and *CTSD* gene expression. However, a negative correlation was observed in Luminal A and Luminal B patients.

The level of *CTSD* expression was elevated in breast cancer tumors when compared to normal tissues, as demonstrated by the differential gene expression in clinical patients. In patients with cathepsin gene expression, there was no significant difference in the ER status. The results indicated that there was a significant increase in the *CTSD* gene expression levels in stages 3 and 4 for all patients with breast cancer. Nonetheless, for subcategories such as Her2, Luminal A, and Luminal B, these levels were significantly expressed in stage 4. In patients with Basal breast cancer, there was no significant difference in the gene expression at any stage.

The results indicated that the E2 levels increased the EGFR protein expression, whereas CUR alone and in the presence of E2 decreased such levels after 48 h. Such an increase could be explained as a response to a signaling pathway enhancing such expression [32]. There was no significant difference between *ESR1* and *EGFR* in every breast cancer subtype. Differential gene expression showed that, in clinical patients, the level of *EGFR* was observed to be greater in healthy tissue as compared to tumor tissue, and patients with a negative ER status were more likely to have a high *EGFR* expression. The results showed that, in breast cancer subtypes such as Her2, Luminal A, and Luminal B, the *EGFR* levels were only significantly expressed in stage 4. In patients with the Basal subtype, no significant gene expression was observed at any stage.

The results revealed that the E2 levels increased the cyclin D1 and *CCND1* gene expression, whereas CUR alone and in the presence of E2 decreased such levels after 48 h. This increase was probably due to a response to a signaling pathway enhancing such expression [32], whereas CUR and its combination with E2 decreased such effects. It is known that the cell-cycle-regulating protein cyclin D1 is encoded by the *CCND1* gene, which influences cell cycle control and accounts for roughly 13% of breast cancers [33]. In patients with the Her2, Luminal A, or Luminal B subtypes, a direct correlation was observed between the expression levels of *ESR1* and *CCND1*. However, this correlation was non-significant in Basal patients. The expression of *CCND1* was found to be more prevalent in breast cancer tumors as compared to normal tissues, as per the differential gene expression seen in clinical patients. Cyclin D1 gene expression was found in breast cancer patients with a positive ER status. Some genes do not present these ERE sites (indirectly regulated genes), such as the gene coding for the cyclin D1 gene, *CCND1*. It is expected that the mRNA levels of ERα-regulated genes would increase in cells exposed to E2 and decrease in those exposed to CUR, alone and in the presence of E2 [31]. The results showed that, in breast cancer subtypes such as Her2, Luminal A, and Luminal B, these levels were only significantly expressed in stage 4. In patients with the Basal subtype, no significant gene expression was observed at any stage.

The results depicted that the E2 levels increased BCL2 and Bax protein expression, whereas CUR alone and in the presence of E2 decreased those levels after 48 h. Such an increase could be explained as a response to a signaling pathway enhancing such expression [32]. The markers associated with apoptosis showed a difference in protein levels, suggesting that CUR induced the cell growth inhibition of Bcl2 and an increase in Bax. The results demonstrated cell death since the cells treated with CUR alone and CUR+E2 combined showed the characteristic morphology of cell death. The results showed that all the breast cancer patients had considerably elevated BCL2 gene expression levels in stages 3 and 4. Nevertheless, the BCL2 gene expression levels in subtypes such as Her2, Luminal A, and Luminal B were only significantly expressed in stage 4. The gene expression levels were not significantly high in any of the stages in Basal patients.

The expression levels of *ESR1* and *BCL2* demonstrated a positive correlation in patients with the Basal, Her2, Luminal A, or Luminal B subtypes. It was observed that the expression of the *BLC2* gene was significantly greater in normal tissues compared to breast cancer tumors in clinical patients who showed variations in gene expression. A positive ER status was observed in all breast cancer patients with *BCL2* gene expression. Some genes do not present these ERE sites (indirectly regulated genes), such as the gene coding for the *BCL2* gene. It is expected that the mRNA levels of ERα-regulated genes would increase in cells exposed to E2 and decrease in those exposed to CUR alone or in the presence of E2 [31].

Previous reports have shown the combined effects of curcumin and chemotherapy drugs [24]. Curcumin and paclitaxel were tested alone and in combination to study cell death in human breast cancer cell lines such as MCF-7, MDA-MB-231, and MCF-10F. In particular, the results showed that curcumin and paclitaxel caused apoptosis and necrosis, as confirmed using multiple methods. However, the combination had a lesser effect on the malignant MDA-MB-231 cell line compared to the MCF-7 or MCF-10F cell lines. It was found that curcumin and paclitaxel together caused more apoptosis than either substance alone in breast cancer cell lines [24].

CUR seemed to regulate the EMT by promoting β-catenin, a marker associated with cell adhesion since it increased in the cells treated with CUR alone or CUR+E2 combined, while Vimentin, a marker of mesenchymal cells, or cells with invasive capabilities decreased under similar conditions. The effects of CUR on genes associated with the EMT were previously assessed [25], where miR-34a was demonstrated to act as a tumor suppressor gene or oncogene that regulates invasion and migration. This research investigated the potential of CUR to inhibit migration and invasion, with a focus on miRNAs as controllers of genes such as rho A, Axl, Slug, and CD24. Rho A plays a role in the processes of migration and invasion, while the Axl, Slug, and CD24 genes contribute to the EMT in the non-malignant MCF-10F and the malignant MDA-MB-231 cell lines. These findings indicate that both cell lines tested negative for estrogen receptors (ERs), the progesterone receptor (PgR), and human epidermal growth factor receptor 2 (HER2), and that CUR regulated genes connected with the EMT via miRNA, independently of their expression [25].

Another study [26] indicated that CUR decreased the EMT through a Pirin-dependent mechanism in cervical cancer cells. Such research showed that viral oncoproteins regulate the expression of Pirin, an oxidative stress sensor that plays a role in EMT and cellular migration. The results showed that exposure to 20 μM CUR reduced migration, EMT, and Slug, Vimentin, and N-cadherin protein expression. According to such results, CUR might decrease the EMT via a Pirin-dependent mechanism. Other authors [34] have worked on the anti-metastasis activity of CUR against breast cancer via the inhibition of stem cell-like properties and the EMT, and they suggested that the inhibitory effects of CUR on breast cancer cells might be connected to the EMT process and resistance to cancer stem-like characteristics.

According to these findings, CUR may have some sort of anti-metastatic properties for breast cancer. Another group [35] found that, in triple-negative breast cancer-bearing mice, CUR ameliorated the muscle malignant metabolic profile, mitochondrial dysfunction, ubiquitination, and inflammation by modulating the NF-*κ*B/UPS axis. This prevented muscle atrophy and loss of function. Moreover, it was found that the ability of CUR to inhibit the TNF-α-induced NF-*κ*B activation of MCF-7 cells lies in its ability to do so through suppressing proteasomal activities rather than I*κ*B kinase activation [36]. On the other hand, a potential therapeutic target for the treatment of breast cancer patients is WZ35, an analog of CUR, which suppresses tumor cell development via the ROS-YAP-JNK signaling pathway [37].

In general, the great molecular complexity that CUR possesses allows it to modulate multiple signaling pathways, such as those involved in cell survival and tumor suppression, the caspase pathway in apoptosis, and death receptor pathways [10]. Considering the interference of this substance with the oncogenic signaling pathways that reactivate ERα, it is important to acknowledge the effect of this substance on ERα activity to consider its therapeutic use.

However, its therapeutic utility is somewhat hampered by its poor absorption in the small intestine and its extensive metabolism in the liver, which impedes its oral bioavailability. To overcome these barriers, several approaches have been devised, such as the use of adjuvants to inhibit the metabolism of curcumin and allow innovative oral delivery mechanisms. It is important to design new techniques to amplify the solubility of curcumin, extend its duration in the plasma, refine its pharmacokinetic characteristics, and boost its cellular absorption, thereby amplifying its therapeutic efficacy, as noted by other authors [38].

Therefore, the pleiotropy of CUR makes this substance a potential multi-target drug in breast cancer and even more so in ERα-positive breast cancer, since it has been demonstrated to modulate oncogenic signaling pathways that could reactivate ERα through a mechanism independent of its ligand binding [39].

It is important to mention that this work has a limitation. Only one cell line was used in this study, which might not be representative of the complexity of luminal breast cancer.

## 5. Conclusions

In summary, this investigation revealed that both CUR and CUR+E2 reduced cell proliferation, impacting the EMT as indicated by β-catenin and Vimentin gene and protein expression. This implies that CUR may influence the metastatic characteristics of cancerous cells and have antitumor effects on breast cancer cells. Therefore, it was demonstrated that a natural agent can inhibit cancer progression and possibly increase drug sensitivity. Patients in stages 3 and 4 of breast cancer subtypes such as Her2, Luminal A, and Luminal B who test positive for *CCND1*, *CTSD*, *EGFR*, *BCL2*, and *ESR1* gene expression might benefit in this regard. Thus, CUR could become a potential natural adjuvant treatment for patients with ERα-positive breast cancer and those with resistance or poor response to endocrine therapy, since the reactivation of ERα is inevitable.

## Figures and Tables

**Table 1:** The order and hybridization temperature of the primers utilized in RT-qPCR.

Gene	Primer Sequence ^a^	Hybridization Temperature [°C] ^b^
*ESR1*	F: CCACCAACCAGTGCACCATT	58.5
	R: GTCTTTCCGTATCCCACCTTT	55.4
*CTSD*	F: GCTACAAGCTGTCCCCAGAG	63.9
	R: CTCTACCCCCACCAAACAGA	63.8
*EGFR*	F: GCGTCTCTTGCCGGAATGT	58.2
	R: GGCTCACCCTCCAGAAGGTT	59.3
*CCND1*	F: AATGACCCCGCACGATTTCA	57.3
	R: TGAGGCGGTAGTAGGACAGG	58.1
*BCL2*	F: TACCTGAACCGGCACCTG	57.2
	R: GCCGTACAGTTCCACAAAGG:	56.2
*ACTB*	F: TGCCGACAGGATGCAGAAG	57.7
	R: GCCGATCCACACGGAGTACT	59.1

^a^ The utilized PCR primer sequence produced an outcome of the specified magnitude, arranged in the 5′→3′ direction. F signifies forward, while R signifies reverse. ^b^ Annealing temperature.

**Table 2:** The clinical significance of genes across multiple breast cancer subtypes was explored through the disease stage factor.

Breast Cancer	*CTSD*	*EGFR*	*CCND1*	*BCL2*
All breast cancer (*n* = 1100)	3, 4 ***	3, 4 ***	3, 4 ***	3, 4 ***
Basal (*n* = 191)	N.S.	N.S.	N.S.	N.S.
Her2 (*n* = 82)	4 *	4 *	4 *	4 **
Luminal A (*n* = 568)	4 ***	4 ***	4 ***	4 ***
Luminal B (*n* = 219)	4 **	4 *	4 **	4 *

The number of stars denotes the statistical significance (as per the Cox proportional hazard model: * signifies a *p*-value < 0.05; ** indicates a *p*-value < 0.01; *** implies a *p*-value < 0.001); 3, 4 are factors of the clinical stage; N.S. stands for non-significant. The data were derived from TIMER2.0 (accessed on 17 June 2022) in breast cancer, reference number [20].

## Data Availability

The pertinent clinical information featured in this study can be freely accessed on TIMER2.0 at http://timer.cistrome.org, with the reference number [20] (retrieved on 6 August 2021); the UCSC Xena online exploration resources can also be freely used at http://xena.ucsc.edu/, with reference number [21] (obtained on 20 August 2021). The data generated in the present study may be requested from the corresponding author.
